# Maintaining Medical Resources to Treat Paediatric Injuries during COVID-19 Lockdown Is Essential—An Epidemiological Analysis of a Level 1 Trauma Centre in Central Europe

**DOI:** 10.3390/ijerph18115829

**Published:** 2021-05-28

**Authors:** Stephan Payr, Andrea Schuller, Theresia Dangl, Philipp Scheider, Thomas Sator, Britta Chocholka, Manuela Jaindl, Elisabeth Schwendenwein, Thomas M. Tiefenboeck

**Affiliations:** Department of Trauma Surgery, University Clinic of Orthopaedics and Trauma Surgery, Medical University of Vienna, 1090 Vienna, Austria; stephan.payr@meduniwien.ac.at (S.P.); n1617891@students.meduniwien.ac.at (A.S.); theresia.dangl@meduniwien.ac.at (T.D.); philipp.scheider@meduniwien.ac.at (P.S.); n1447277@students.meduniwien.ac.at (T.S.); britta.chocholka@meduniwien.ac.at (B.C.); manuela.jaindl@meduniwien.ac.at (M.J.); elisabeth.schwendenwein@meduniwien.ac.at (E.S.)

**Keywords:** paediatric trauma, fractures, traumatic brain injury, COVID-19, pandemic

## Abstract

Background: This study examined the effect of the COVID-19 pandemic and the resulting decrease in the incidence of various categories of injuries, with the main focus on fractures and mild traumatic brain injuries in a paediatric population. Methods: This retrospective cohort study evaluated all children from 0 to 18 years of age presenting with an injury at the level 1 trauma centre of the University Clinic of Orthopaedics and Trauma Surgery in Vienna during the lockdown from 16 March to 29 May 2020 compared to records over the same timeframe from 2015 to 2019. Results: In total, 14,707 patients with injuries were included. The lockdown did not lead to a significant decrease in fractures but, instead, yielded a highly significant increase in mild traumatic brain injuries when compared to all injuries that occurred (*p* = 0.082 and *p* = 0.0001) as well as acute injuries (excluding contusions, distortions and miscellaneous non-acute injuries) (*p* = 0.309 and *p* = 0.034). Conclusions: The percentage of paediatric fractures did not decrease at the level 1 trauma centre, and a highly significant proportional increase in paediatric patients with mild traumatic brain injuries was observed during the COVID-19 lockdown. Therefore, medical resources should be maintained to treat paediatric trauma patients and provide neurological monitoring during pandemic lockdowns.

## 1. Introduction

The World Health Organization (WHO) declared the COVID-19 outbreak a pandemic on 11 March 2020 [1].

Several public health measures, such as social distancing, wearing masks in public, prolonged school closures, and the cancellation of sporting activities, were implemented in countries all over the world. In Austria, this type of lockdown (LD) was announced on 16 March 2020 [2,3]. Public health measures (limitations on daily activities and an order to stay at home) are intended to avoid or limit the spread of the virus. Such restrictions can affect the epidemiology of patients coming to the emergency department.

In Austria, schools were re-opened in mid-May, and sporting activities, sport clubs and lido were re-opened on 29 May 2020, representing the end of the first LD [2,4].

Studies show a decrease in fracture incidence among children and adolescents who are affected by school closures alongside a reduction in after-school activities [1,5,6,7]. Therefore, it was suggested to redeploy trauma surgeons to other clinical departments during the pandemic [1]. Currently, many studies have explored fractures as acute injuries in children during the pandemic LD [1,6,7,8]. At the beginning of this LD, schools, sports and other public activities ceased, and we observed a sudden reduction in children with injuries presenting to our trauma department. However, injuries may still occur and could potentially increase at home during LD, a period in which parents must balance their work at home with watching their children. Therefore, in this study, we aimed to evaluate additional categories of paediatric injuries (mild traumatic brain injury, wounds and more) during LD. This study especially focuses on the category of mild traumatic brain injuries (mTBIs). It was previously noted that the treatment and incidence of mTBIs have underestimated clinical importance and should be regarded as an important health care issue due to the large patient population that is affected [9,10]. The objective of this study was to evaluate how the COVID-19 pandemic, leading to the lockdown from 16 March to 29 May 2020, affected the incidence and characteristics of paediatric injuries presenting to a single level 1 trauma centre in Central Europe compared to the injuries observed over the same periods of time in the previous five years (2015–2019). We hypothesised that fracture incidence would decrease during lockdown due to the strict stay-at-home policy implemented by the government, whereas paediatric patients with mild traumatic brain injuries (mTBIs) would increase at the trauma department during lockdown for the same reasons.

## 2. Materials and Methods

This retrospective study was approved by the Ethics Committee of the Medical University of Vienna (Code: 2315/2020). In this study, we included all children from 0 to 18 years of age who presented with an injury at the trauma department of the University Clinic of Orthopaedics and Trauma Surgery during the lockdown from 16 March to 29 May 2020 compared to the same period of time in 2015–2019. The Austrian lockdown period (16 March until 29 May 2020) refers to the period of restrictions that were implemented by the government; these restrictions were also monitored by the police. Such restrictions included school closures, the cancellation of sporting activities and a stay-at-home policy. All patient data were extracted from the patients’ charts stored in the information management system (AKIM) of the General Hospital of Vienna.

Demographic variables (sex and patient age at injury) were collected. Patients’ injuries were categorised from most to least acute according to their primary diagnoses.

Fracture characteristics were divided into upper extremity (UE) or lower extremity (LE) fractures as well as axial skeleton fractures (including those of the skull, spine, ribs, scapula and pelvis) and treatment modalities (operative vs. conservative).

The rest of the injuries were categorised as mTBIs (including contusions and concussions), wounds (including lacerations, bites, cuts, stabs, skin defects and subtotal or total amputations), contusions, distortions and miscellaneous. “Miscellaneous” was further divided in “miscellaneous acute” and “miscellaneous not acute”. “Miscellaneous acute” was defined as injuries pain, inhibition of the range of motion and a lack of function including pronation dolorosa, dislocated joints, lesions or the rupture of a ligament. “Miscellaneous non-acute” included injuries with pain and only included injuries such as superficial grazing, local hematoma with the intact skin, pain with inadequate trauma or dental defects. Acute injuries were defined as injuries that needed examination and treatment as soon as possible, with the potential for consequent impairment if not treated, such as fractures, mTBIs, wounds, dislocated joints and lesions of the ligaments or tendons.

Injuries that occurred during the COVID-19 outbreak restrictions were further grouped together as a cohort referred to as the “lockdown” (LD) group. Those in the LD group were compared to those with injuries who presented to the care centre during the 2015–2019 study windows, referred to as the “pre-lockdown” (pre-LD) group. A comparison was conducted between fractures and mTBIs in the LD group and the pre-LD group.

A comparison period of five years was chosen to minimise potential bias by ensuring an adequate sample size for the control. Further, in these years, no similar restrictions were implemented at any time, making this timeframe adequate as a control period.

Descriptive data (mean ± SD/percentages) are reported for the entire patient cohort. Differences between the means and proportions were tested with a Fisher exact test for categorical variables and an unpaired *t*-test for continuous variables; when the results were not normally distributed, a Mann–Whitney U-test was used. Normal distribution was tested using a Shapiro–Wilk test.

Statistical significance was set at a level of *p* < 0.05. Microsoft Excel and the GraphPad software version 6.00 (GraphPad Software, San Diego, CA, USA) were used for statistical analysis.

## 3. Results

**Figure 1:**
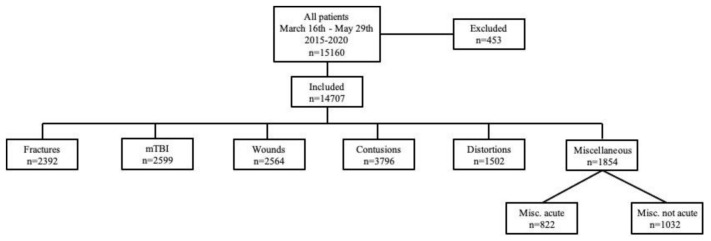
Flow chart of the overall study population according to categorised injuries.

In total, 14,707 injured children were included in the study. First, 453 patients were excluded; the reasons for exclusion included administrative errors, patients who left before their medical examinations and patients who did not have a traumatological primary diagnosis and were, therefore, transferred to other specialists. A general overview of the study population is given in Figure 1. Injuries during the observation period categorised by year are given in Table 1. Table 2 shows the average numbers and percentages of each category for the entire pre-LD period and further presents comparisons (including *p*-values) of each category in proportion to all injuries between the pre-LD and LD period. Notably, the absolute numbers were generally reduced in every category of injury during LD. Regarding the decrease in absolute numbers, only one-third of all patients in the pre-LD period had an acute injury compared to two-thirds of patients during LD. Therefore, a significant reduction in non-acute injuries during LD was observed (*p* = 0.0001).

### 3.1. Fractures during LD vs. the Pre-LD Period

During LD, 158 fractures (18.7%; 98 m, 60 f, mean age: 8.2 ± 5) occurred among a total number of 844 injuries. These fractures included 116 fractures of the UE (upper extremity), 30 of the LE (lower extremity) and 12 of the axial skeleton (Table 3). In total, 96 (61 male, 35 female) of the fractures of the UE were treated conservatively, and 20 (11 m, 9 f) were treated operatively; 27 (14 m, 13 f) fractures of the LE were treated conservatively, and 3 (2 m, 1 f) were treated operatively.

The mean of the pre-LD period revealed a mean of 447 fractures (mean 16.1%, 277 m, 170 f, mean age: 9.8 ± 4.6) from a mean total number of 2773 injuries. A mean of 314 fractures (199 m, 115 f) affected the UE, 110 affected the LE (63 m, 47 f), and 23 affected the axial skeleton (16 m, 7 f) (Table 4). A mean of 277 fractures (174 m, 103 f) of the UE were treated conservatively, and a mean of 37 (25 m, 12 f) were treated operatively. Concerning the mean of the LE, 102 fractures (58 m, 44 f) were treated conservatively, and eight (5 m, 3 f) were treated operatively; 22 fractures (15 m, 7 f) of the axial skeleton were treated conservatively, and one was treated operatively.

### 3.2. mTBIs during LD vs. the Pre-LD Era

During LD, 212 mTBIs (126 m, 86 f, mean age: 4.1 ± 4.8) represented 25.1% of the total number of injuries (Table 5).

The mean of the pre-LD period revealed a mean of 477 mTBIs (266 m, 211 f, mean age: 5.4 ± 5.3), representing a mean of 17.2% of the total injuries.

### 3.3. Wounds during LD vs. the Pre-LD Era

During LD, 188 wounds (115 m, 73 f, mean age: 6.3 ± 5.3) represented 22.3% of all injuries (Table 6); seven needed surgery and 181 were managed with standard wound management (including wound cleaning and stitches or only plaster/bandages when appropriate).

The majority were lacerations, with 130 and 123 (94.6%) affecting the head. The rest included six bites (three human/three animal), 37 cuts, one amputation, three skin defects and 11 stab wounds.

The mean of the pre-LD period revealed a mean of 475 wounds (mean 17.1%, 292 m, 182 f, mean age: 7.1 ± 5.5) from a total number of 2773 injuries. A mean of eight needed surgery, and 467 received standard wound management (as described above). A mean of 327 lacerations occurred, 283 (86.4%) of which affected the head. Furthermore, there was a mean of 39 bites (12 human/27 animal), 83 cuts, one amputation, seven skin defects and 27 stab wounds.

**Figure 2:**
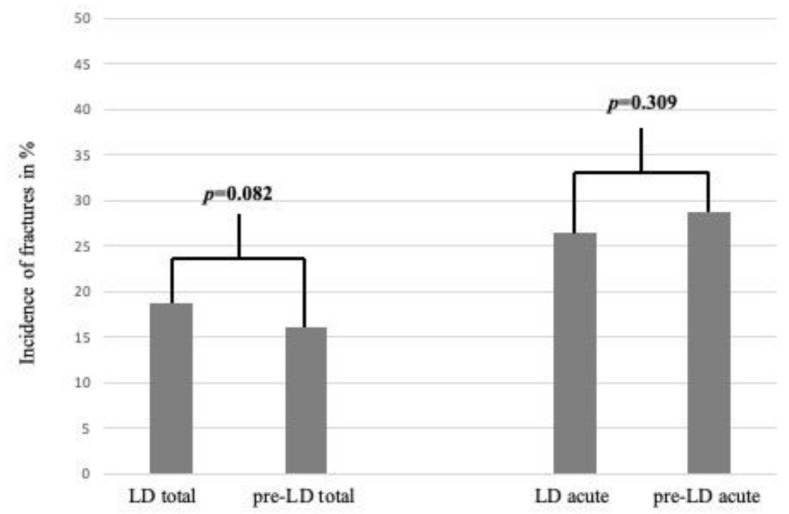
Comparison of fractures in % during LD and pre-LD periods. “LD total” and “pre-LD total” include the total numbers of injuries, and “LD acute” and “pre-LD acute” include only the acute injuries (fractures, mTBIs, wounds and miscellaneous acute).

Comparing the incidence of fractures (LD vs. pre-LD) as a proportion of the total number of injuries, no statistical difference was observed (*p* = 0.082) (Figure 2).

**Figure 3:**
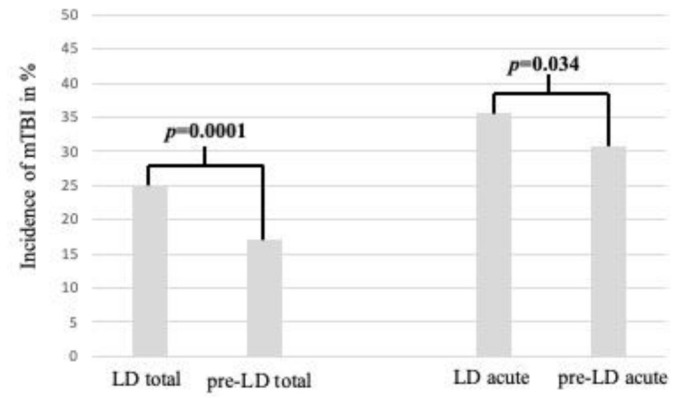
Comparison of mTBIs in % during the LD and pre-LD periods. “LD total” and “pre-LD total” include the total numbers of injuries, and “LD acute” and “pre-LD acute” include only the acute injuries (fractures, mTBIs, wounds and miscellaneous acute).

By comparing the incidence of mTBsI (LD vs. pre-LD) as a proportion of the total number of injuries, a highly significant increase was observed (*p* = 0.0001) (Figure 3).

**Figure 4:**
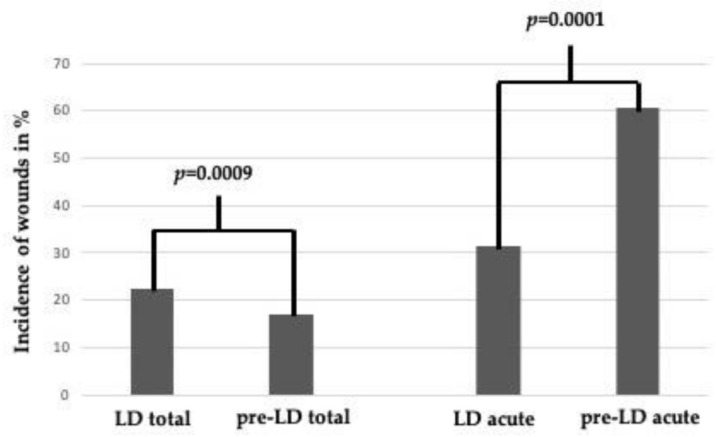
Comparison of wounds in % during LD and the pre-LD periods. “LD total” and “pre-LD total” include the total numbers of injuries, and “LD acute” and “pre-LD acute” include only the acute injuries (fractures, mTBIs, wounds and miscellaneous acute).

By comparing the incidence of wounds (LD vs. pre-LD) as a proportion of the total number of injuries, a significant increase was observed (*p* = 0.0009) (Figure 4). When setting the incidence of fractures and mTBIs as a proportion of only acute injuries (only fractures, mTBIs, wounds and miscellaneous acute) instead of the total number of injuries (including contusions, distortions and miscellaneous non-acute), a significant increase in mTBIs were still observed among the children (*p* = 0.034), but no statistical difference in the incidence of fractures (*p* = 0.309) was found. Compared to the acute patients, there was a highly significant decrease in wounds during the LD period (*p* = 0.0001). However, the percentage of lacerations affecting the head compared to all lacerations was significantly higher during LD (*p* = 0.013). The children with mTBIs were significantly younger during LD than those in the pre-LD period (mean age: 4.1 ± 4.8 vs. 5.4 ± 5.3; *p* = 0.006). The incidence of severe TBIs (skull fractures and brain haemorrhages) did not significantly differ between LD and the pre-LD periods (*p* = 0.165 and *p* = 0.233) or when only compared to acute injuries (*p* = 0.331 and *p* = 0.277). Finally, among all the injuries, children with non-acute injuries (contusions and distortions) were significantly less common during lockdown compared to the previous years (*p* = 0.0001).

## 4. Discussion

The main findings indicate that the absolute numbers of patients with paediatric injuries visiting the ED decreased during LD. This is in accordance with the current literature [1,5,6,7]. However, when taking a closer look at the LD numbers, the fractures did not decrease significantly in proportion to all injuries compared to previous time periods, which contrasts with the literature. The numbers in this study indicate that two fractures, three mTBIs and three wounds per day (mean) presented at the level 1 trauma department during the strict 68 day LD. In the LD cohort, we observed a significant proportional increase in mTBIs in the paediatric population, as hypothesised. This proportional increase was significant compared to all injuries as well as when compared to only acute injuries. Additionally, the wounds revealed a significant proportional increase in the paediatric population during LD. However, when taking into account only the acute injuries, wounds significantly decreased during the LD period. This number of wounds must be put into perspective, since two-thirds of the patients visiting the ED under non-pandemic conditions did not show any acute injuries, whereas during LD, only one-third of the presenting patients had non-acute injuries.

Lacerations affecting the head were observed in over 90% of patients during the LD period. This result supports the findings of this study showing a higher risk of mTBIs and head injuries in paediatric patients presented at the ED during LD. The present study is the first of its kind to analyse various categories of paediatric injuries during the pandemic LD while focusing on mTBIs. To date, the literature has solely focused on paediatric fractures during the COVID-19 pandemic [1,7,11,12,13]. Indeed, the decrease/increase in injuries during the COVID-19 pandemic is still controversial in the literature. There are several studies showing a reduction in fractures after the first LD [1,5,7,12]. Despite these observations, Hernigou et al. showed a decrease in the total numbers of injuries; however, the proportion of injured children increased [8]. Another study observed a significant increase in fractures during the COVID-19 pandemic accompanied by a decrease in overall paediatric visits at their department but lacking any signs of significance [11]. This decrease/increase in paediatric injuries agrees with the regional differences and strictness of the COVID-19 LD policies [14]. This could also be the reason for the observations in the present study. Further analysis of the data showed that fewer patients (in total and in relation to all injuries) presented injuries that were not necessarily acute (contusions, distortion) during the COVID-19 pandemic, as mentioned above. This result might reflect a certain awareness during the COVID-19 pandemic to avoid potential viral exposure during hospital visits. However, the literature suggests that the time to presentation with fractures did not differ among paediatric patients [1]. This fact suggests that when children complained about severe pain during the pandemic, parents presented rapidly to the trauma department despite COVID-19, rather than risk the health of their children.

The second main finding of this study was the significant increase in mTBIs during the LD. Analysis of mTBIs is of importance for parents’ daily routines when introducing stay-at-home policies. One reason for the increased percentage of infants sustaining mTBIs during LD may be parents working from home. Parents working from home experience increased stress due to the fact of parenting their children while working and being unable to unplug from work [15]. Furthermore, the observation of a decrease in age (from 5 to 4) among infants suffering from mTBIs coincides with the fact that it is especially difficult for parents with younger children (0–5 years) to balance work and family during a pandemic [16]. In this study, 76% of the mTBIs occurred at home, thereby supporting this theory. In children > 5 years of age, the rate of mTBIs decreased, which might be due to the reduced engagement in sports and outdoor activities [6]. Focusing on mTBIs is crucial: although a single mTBI is a recoverable injury, it still has the potential to yield subsequent injuries [9,17]. Notably, repetitive mild traumatic brain injuries (RmTBIs) can lead to consequential damage, greater symptoms, and prolonged recovery, and the paediatric population is at significant risk of sustaining RmTBIs [17,18]. The consequences of LD and work at home—and, therefore, the increase in RmTBIs during this period—require further studies to confirm.

### Limitations

The study settings during the COVID-19 pandemic were similar in all countries, thereby producing similar conclusions around the world [1,6,8,19,20]. Admissions at regional major trauma centres and the number of acute surgeries remained relatively stable in relation to overall patient visits [6,20,21]. This study had several limitations such as its retrospective single-centre design. However, the data in this study were obtained from the largest university hospital in the world and the main paediatric trauma centre in Austria. This fact might have led to a disproportionate increase in cases, as several trauma departments in Vienna had to drastically reduce their capacities. This fact, however, cannot be proven. Instead, we focused on the primary diagnosis when categorizing injuries retrospectively. This retrospective method was chosen to simplify the evaluation due to the massive amount of data arising over five years of comparison (a longer period of comparison than that in other studies) [1,6,7,11,12,13,19,20]. Many studies compare their data to the data of the most recent year, making their cohorts and, thus, their results, relatively susceptible to bias [6,8,20,21]. We included a longer period of comparison and a larger number of patients compared to the numbers in the current literature. Because of this massive quantity of data, certain accompanying injuries might have been missed. However, the results nevertheless reflect the actual patient numbers during the studied periods. This study, therefore, gives an accurate overview of fractures and mTBIs from a level 1 trauma centre, unlike the majority of current data that come from tertiary trauma centres [1,7,11,12,13].

## 5. Conclusions

Despite lower absolute numbers of injuries, the percentage of paediatric fractures did not decrease at this level 1 trauma centre. In addition, a highly significant proportional increase in paediatric patients with mTBIs was observed during the COVID-19 LD.

Parents should be aware of the higher risk of mTBIs in children between 0 and 5 years of age at home during LD situations. During LD, doctors at the ED had a greater chance of encountering and treating acute injuries among children.

Due to the lower patient numbers, the centralization of paediatric trauma is recommended during times of a pandemic, and a trauma surgeon with paediatric trauma experience should stay on call at these centres.

## Figures and Tables

**Table 1:** Overview of the injuries categorised according to the year of observation.

2020	n=	% of All Injuries	Male	Male Mean Age ± SD	Female	Female Mean Age ± SD	Total Mean Age ± SD
			n/%		n/%		
Total	844	100	480/100%	6.9 ± 5.9	364/100%	6.7 ± 5.4	6.9 ± 5.7
Fractures	158	18.7	98/20.4%	8.3 ± 5.1	60/16.5%	8.1 ± 4.9	8.2 ± 5.0
mTBIs	212	25.1	126/26.3%	4.3 ± 5.1	86/23.6%	3.7 ± 4.5	4.1 ± 4.8
Wounds	188	22.3	115/24.0%	5.8 ± 4.8	73/20.1%	7.1 ± 5.9	6.3 ± 5.3
Contusions	132	15.6	66/13.8%	8.2 ± 5.9	66/18.1%	6.8 ± 5.8	8.4 ± 5.8
Distortions	50	5.9	25/5.2%	13.8 ± 3.8	25/6.9%	12.4 ± 4.0	13.1 ± 3.9
Miscellaneous	104	12.3	50/10.4%	7.2 ± 6.0	54/14.8%	6.2 ± 5.5	6.7 ± 5.8
Miscellaneous acute	39	4.6	17/3.5%	8.6 ± 6.8	22/6.0%	6.9 ± 6.3	7.7 ± 6.7
Miscellaneous non-acute	65	7.7	33/6.9%	6.4 ± 5.3	32/8.8%	5.8 ± 4.9	6.1 ± 5.1
**2019**	**n=**	**% of All Injuries**	**Male**	**Male Mean Age ± SD**	**Female**	**Female Mean Age ± SD**	**Total Mean Age ± SD**
			n/%		n/%		
Total	2736	100	1555/100%	8.9 ± 5.7	1181/100%	8.6 ± 5.6	8.8 ± 5.6
Fractures	424	15.5	247/15.9%	10.3 ± 4.7	177/15.0%	9.0 ± 4.6	9.8 ± 4.7
mTBIs	514	18.8	274/17.6%	5.5 ± 5.4	240/20.3%	5.6 ± 5.5	5.5 ± 5.4
Wounds	440	16.1	264/17.0%	6.9 ± 5.4	1761/14.9%	7.2 ± 5.5	7.0 ± 5.4
Contusions	720	26.3	428/27.5%	10.1 ± 5.3	292/24.7%	10.7 ± 5.0	10.3 ± 5.2
Distortions	274	10	147/9.5%	12.5 ± 4.5	127/10.8%	12.2 ± 4.1	12.4 ± 4.3
Miscellaneous	364	13.3	195/12.5%	8.7 ± 5.8	169/14.3%	8.0 ± 5.7	8.4 ± 5.8
Miscellaneous acute	153	5.6	70/4.5%	10.3 ± 5.5	83/7.0%	9.4 ± 5.8	9.9 ± 5.7
Miscellaneous non-acute	211	7.7	125/8.0%	7.9 ± 5.7	86/7.3%	6.5 ± 5.2	7.3 ± 5.6
**2018**	**n=**	**% of All Injuries**	**Male**	**Male Mean Age ± SD**	**Female**	**Female Mean Age ± SD**	**Total Mean Age ± SD**
			**n/%**		**n/%**		
Total	2684	100	1530/100%	8.5 ± 5.6	1154/100%	8.3 ± 5.5	8.4 ± 5.5
Fractures	496	18.5	294/19.2%	9.4 ± 4.8	202/17.5%	8.8 ± 4.3	9.2 ± 4.6
mTBIs	464	17.3	264/17.3%	5.2 ± 5.0	200/17.3%	4.7 ± 5.1	5.0 ± 5.0
Wounds	438	16.3	278/18.2%	7.0 ± 5.3	160/13.9%	7.2 ± 5.5	7.1 ± 5.4
Contusions	652	24.3	360/23.5%	9.4 ± 5.2	292/25.3%	9.5 ± 5.1	9.5 ± 5.2
Distortions	286	10.7	140/9.2%	12.3 ± 4.9	146/12.7%	12.7 ± 4.2	12.5 ± 4.6
Miscellaneous	348	13	194/12.7%	8.9 ± 5.9	154/13.3%	6.9 ± 5.6	8.0 ± 5.9
Miscellaneous acute	154	5.7	79/5.2%	12.7 ± 3.9	75/6.5%	12.6 ± 4.0	8.9 ± 5.9
Miscellaneous non-acute	194	7.2	115/7.5%	7.9 ± 6.0	79/6.8%	7.5 ± 5.2	7.3 ± 5.7
**2017**	**n=**	**% of All Injuries**	**Male**	**Male Mean Age ± SD**	**Female**	**Female Mean Age ± SD**	**Total Mean Age ± SD**
			**n/%**		**n/%**		
Total	2778	100	1641/100%	9.1 ± 5.5	1137/100%	8.4 ± 5.7	8.8 ± 5.6
Fractures	466	16.8	305/18.6%	10.4 ± 4.6	161/14.2%	8.5 ± 4.3	9.7 ± 4.6
mTBIs	495	17.8	288/17.6%	5.8 ± 5.4	207/18.2%	4.7 ± 5.2	5.3 ± 5.3
Wounds	439	15.8	278/16.9%	7.4 ± 5.6	161/14.2%	6.6 ± 5.4	7.1 ± 5.5
Contusions	757	27.3	439/26.8%	10.3 ± 5.0	318/28.0%	9.4 ± 5.4	9.9 ± 5.2
Distortions	289	10.4	149/9.1%	12.5 ± 4.4	140/12.3%	12.9 ± 4.0	12.7 ± 4.2
Miscellaneous	332	12	182/11.1%	9.4 ± 5.8	150/13.2%	8.7 ± 6.0	9.0 ± 5.9
Miscellaneous acute	173	6.2	94/5.7%	10.5 ± 5.4	79/6.9%	9.2 ± 5.8	9.9 ± 5.7
Miscellaneous non-acute	159	5.7	88/5.4%	8.3 ± 5.8	71/6.2%	8.0 ± 6.0	8.2 ± 5.9
**2016**	**n=**	**% of All Injuries**	**Male**	**Male Mean Age ± SD**	**Female**	**Female Mean Age ± SD**	**Total Mean Age ± SD**
			**n/%**		**n/%**		
Total	2931	100	1697/100%	9.4 ± 5.6	1234/100%	8.5 ± 5.6	9.0 ± 5.6
Fractures	442	15.1	283/16.7%	10.9 ± 4.4	159/12.9%	8.9 ± 4.6	10.2 ± 4.6
mTBIs	498	17	274/16.1%	6.0 ± 5.4	224/18.2%	5.5 ± 5.4	5.8 ± 5.4
Wounds	499	17	309/18.2%	6.6 ± 5.4	190/15.4%	6.5 ± 5.3	6.6 ± 5.3
Contusions	787	26.9	451/26.6%	10.7 ± 5.2	336/27.2%	9.7 ± 5.3	10.2 ± 5.5
Distortions	355	12.1	192/11.3%	13.0 ± 4.1	163/13.2%	12.0 ± 4.2	12.6 ± 4.2
Miscellaneous	350	11.9	188/11.1%	9.8 ± 5.7	162/13.1%	8.4 ± 6.0	9.2 ± 5.9
Miscellaneous acute	153	5.2	85/5.0%	10.1 ± 6.0	68/5.5%	9.5 ± 5.4	9.8 ± 5.8
Miscellaneous non-acute	197	6.7	103/6.1%	9.7 ± 5.3	94/7.6%	7.5 ± 6.2	8.7 ± 5.9
**2015**	**n=**	**% of All Injuries**	**Male**	**Male Mean Age ± SD**	**Female**	**Female Mean Age ± SD**	**Total Mean Age ± SD**
			**n/%**		**n/%**		
Total	2734	100	1554/100%	9.1 ± 5.6	1180/100%	8.7 ± 5.8	8.9 ± 5.7
Fractures	406	14.9	257/16.5%	10.4 ± 4.8	149/12.6%	10.2 ± 4.4	10.3 ± 4.6
mTBIs	416	15.2	230/14.8%	5.7 ± 5.5	186/15.8%	5.4 ± 5.7	5.6 ± 5.6
Wounds	560	20.5	334/21.5%	7.1 ± 5.5	226/19.2%	8.4 ± 6.0	7.6 ± 5.7
Contusions	748	27.4	418/26.9%	10.1 ± 5.1	330/28.0%	9.6 ± 5.5	9.9 ± 5.3
Distortions	248	9.1	131/8.4%	12.5 ± 4.5	117/9.9%	12.1 ± 4.6	12.3 ± 4.6
Miscellaneous	356	13	184/11.8%	10.1 ± 5.9	172/14.6%	8.9 ± 6.0	9.6 ± 6.0
Miscellaneous acute	150	5.5	69/4.4%	11.1 ± 5.5	81/6.9%	9.1 ± 6.1	10.0 ± 5.9
Miscellaneous non-acute	206	7.5	115/7.4%	9.5 ± 6.1	91/7.7%	8.7 ± 5.8	9.2 ± 6.0

**Table 2:** Average numbers and percentages of the pre-LD period plus the *p*-values of each category (pre-LD vs. LD period) in proportion to all injuries.

Average of Pre-LD Period (2015–2019)	n=	% of All Injuries	LD Era	n=	% of All Injuries	*p*-Value	*p*-Value (Compared to Only Acute Injuries)
Total	2773	100	Total	844	100		
Fractures	447	16.2	Fractures	158	18.7	0.082	0.309
mTBIs	477	17.2	mTBIs	212	25.1	0.0001	0.034
Wounds	475	17.1	Wounds	188	22.3	0.0009	0.0001
Contusions	733	26.4	Contusions	132	15.6	0.0001	-
Distortions	290	10.5	Distortions	50	5.9	0.0001	-
Miscellaneous	350	12.6	Miscellaneous	104	12.3	0.858	-
Miscellaneous acute	157	5.6	Miscellaneous acute	39	4.6	-	-
Miscellaneous non-acute	967	7.0	Miscellaneous non-acute	65	7.7	-	-

**Table 3:** Distribution of fractures according to region, sex, and treatment modality during LD.

Fractures in LD (2020)	Total	m/f	Op	m/f	Conservatively	m/f
	158	97/61	23/14.6%	13/10	135/85.4%	84/51
Upper Extremity	116/73.4%	72/44	20/17.2%	11/9	96/82.8%	61/35
Lower Extremity	30/19%	16/14	3/10%	2/1	27/90%	14/13
Axial Skeleton	12/7.6%	9/3	0	-/-	12	9/3

f—female, m—male, Op—operatively.

**Table 4:** Distribution of fractures according to region, sex and treatment modality in the pre-LD period.

Fractures Pre-LD (2015–2019)	Total	m/f	Op	m/f	Conservatively	m/f
	447	277/170	46/10.3%	30/15	401/89.7%	247/154
Upper Extremity	314/70.2%	199/115	37/11.8%	25/12	277/88.2%	174/103
Lower Extremity	110/24.6%	63/47	8/7.3%	5/3	102/92.7%	58/44
Axial Skeleton	23/5.1%	16/7	1/4.3%	-/-	22/95.7%	15/7

f—female, m—male, Op—operatively.

**Table 5:** Distribution of mTBIs during LD and the pre-LD period.

	mTBIs	m/f
LD (2020)	212	126/86
Pre-LD (2015–2019)	477	266/211

f—female, m—male.

**Table 6:** Distribution of wounds during LD and the pre-LD period.

Wounds	Total	Laceration (of the Head n= and in %)	Bite (Human/Animal)	Cut	Amputation	Skin Defect	Stab	Op/Conservative Wound Management	m/f	Mean Age
LD (2020)	188	130 (123; 94.6%)	6 (3/3)	37	1	3	11	7/181	115/73	6.3 ± 5.3
Mean Pre-LD (2015–19)	475	327 (283, 86.4%)	34(6/23)	83	1	7	27	8/467	292/182	7.1 ± 5.5

f—female, m—male, n—number, Op—operatively.

## Data Availability

Data are available from the first author upon request.
